# Effects of short chain fructo-oligosaccharides on selected skin bacteria

**DOI:** 10.1038/s41598-022-13093-5

**Published:** 2022-06-11

**Authors:** Cindy Le Bourgot, Claire Meunier, Elisa Gaio, Vincent Murat, Marta Micheletto, Erik Tedesco, Federico Benetti

**Affiliations:** 1 Tereos, R&D Department, Rue de Senlis, 77290 Moussy-Le-Vieux, France; 2 ECSIN-ECAMRICERT SRL Laboratory, Corso Stati Uniti, 4, 35127 Padua, Italy

**Keywords:** Cell biology, Microbiology, Physiology

## Abstract

The human skin microbiota plays a key role in the maintenance of healthy skin, ensuring protection and biological barrier by competing with pathogens and by closely communicating with the immune system. The development of approaches which preserve or restore the skin microbiota represents a novel target for skincare applications. Prebiotics could be applied to balance almost any microbial community to achieve advantageous effects. However, information about their effectiveness as skin microbiota modulators is limited. The objective of the current study was to evaluate the effects of short chain fructo-oligosaccharides (scFOS) from sugar beet (DP 3–5), well-recognised prebiotics, on some representative bacterial strains of the skin microbiota. We measured the growth and competitive activity of these specific bacteria for the use of scFOS as energy source in minimal medium and in a reconstructed human epithelium (RHE) in vitro model. In minimal growth medium, scFOS promoted and sustained the growth of *Staphylococcus epidermidis* up to 24 h, considered a beneficial skin commensal bacterium, while inhibiting both *Cutibacterium acnes* and *Staphylococcus aureus* growth, regarded as opportunistic pathogens. *S. epidermidis* showed the highest colonization potential and 1% scFOS was effective in shifting the competition in favour of *S. epidermidis* with respect to *C. acnes* in the RHE model. This latter effect was observed following 24 h of exposure, suggesting a long-term effect of scFOS in a highly skin dynamic environment. Therefore, scFOS could be effectively implemented in skincare formulations for recovering skin microbiota homeostasis.

## Introduction

The skin is a complex and dynamic ecosystem representing the largest organ in the human body. Besides the physical barrier, the skin-resident microbiota guarantees protection and a biological barrier by competing with pathogens and by communicating closely with cells and components of the immune system. Therefore, the skin microbiota can be considered as an essential player for the maintenance of a healthy skin^1^. The skin microbiota includes resident microorganisms routinely found in the skin and that are considered to be commensal, meaning that they are usually harmless and most probably provide some benefits to the host^2,3^. Among resident species of the skin microbiota, there are Propionibacteria like *Propionibacterium acnes* also named *Cutibacterium acnes*, coagulase-negative staphylococci like *Staphylococcus epidermidis*, and other types like Corynebacteria, Micrococci and Acinebacter. *S. epidermidis* is a commensal bacterium, being part of the normal skin microbiota that serves to protect human skin from infections and other environmental insults^4^, while *C. acnes* has been linked to acne and associated with many skin pathologies^5,6^. The skin microbiota also includes transient microorganisms, such as *Staphylococcus aureus*, *Escherichia coli* and *Pseudomonas aeruginosa*^4,7^. *S. aureus* has been identified as an important pathogen when over-colonizing the skin, and it is involved in human skin diseases development^2^. Both composition and abundance of the skin microbiota vary considerably according to the parts of the body, between individuals and over time, resulting in an extremely dynamic bacterial community^8,9^.

The human skin microbiota has recently become a focus for both dermatological and cosmetic fields. Understanding the skin microbiota and the way to maintain its delicate balance are essential steps to gain insight into the mechanisms responsible for healthy skin and its appearance. Imbalances in the skin microbiota composition, also named dysbiosis, are associated with several skin pathologies such as acne, eczema or allergies, as well as non-pathological conditions namely sensitive skin, irritation or dry skin. Therefore, the development of approaches which preserve or restore the balance of the microbiota represents a novel target to favour skin health.

While skin microbiota research is in an early stage, evidence suggests that there are many ways to be proactive about skin health, and more particularly about skin microbiota^5,10,11^. Indeed, an increasing number of skin care products started to incorporate prebiotics, with considerable challenges ahead but also many opportunities. While the concept of prebiotics is well known for food product, in which they are used to promote the growth of beneficial bacteria of the gut microbiota, inducing subsequent health benefits^12^, they could be applied to balance almost any microbial community. As such, prebiotics could be used as modulators of the skin microbiota composition to achieve advantageous effects^4^. There are rather small number of studies evidencing the effectiveness of prebiotics to modulate skin microbiota although several reviews raise their potential^4,7,13–15^.

Among recognized prebiotics, short chain fructo-oligosaccharides (scFOS) are well-known for their prebiotic properties on gut microbiota and their benefits for the human health. This prebiotic scFOS (FOSbeauty^®^) is a unique active ingredient obtained from sugar beet sucrose through an enzymatic reaction, with a proprietary enzyme, leading to very short-chain structure (degree of polymerisation comprised between 3 and 5). These are composed of a terminal glucose molecule linked to fructose molecules by a β-1,2-glycosidic bond with a consistent and guaranteed composition, with 37 ± 6% GF2, 47 ± 6% GF3 and 16 ± 6% GF4. Once applied in cosmetic formulations, their effects on the skin microbiota composition are still unknown and they need to be investigated in detail. Therefore, the impact of scFOS on the growth of some bacterial strains, representative of the human skin microbiota, has been evaluated.

## Methods

### Experimental trial

The present study was aimed at evaluating the effects of scFOS (FOSbeauty^®^, Beghin-Meiji) on the in vitro growth and competitive activity of bacterial strains representative of the human skin microbiota (*Staphyloccocus epidermidis*, *Cutibacterium acnes* and *Staphylococcus aureus*).

### Bacterial strains

*Staphyloccocus epidermidis* (ATCC^®^ 12228) and *S. aureus* (ATCC^®^ 6538) were acquired from the American Type Culture Collection (ATCC), while *C. acnes* (CCUG 1794T) was acquired from the Culture Collection University of Gothenburg (CCGU). Bacterial strains, provided in freeze-dried form, were resuspended and inoculated in sterile tubes containing their respective selective medium [tryptic soy broth (TSB) for *Staphylococcus* strains, and TSB + 5% defibrinated sheep blood for *C. acnes* (BTSB)], and left to grow at the optimal condition for each bacterium (i.e. 24 h at 37 °C in aerobic condition for *Staphylococcus* strains, and 48 h at 37 °C in anaerobiosis for *C. acnes*). The resulting bacterial suspension were stored at − 80 °C, following the freezing procedure indicated by the cell banks. At the beginning of the experimental phase, a vial of each bacterium was collected from − 80 °C, partially thawed and a small aliquot collected with a sterile loop and streaked on selective medium-based agar plates. Streaked bacteria were then left to grow in optimal conditions for each strain and, once visible colonies were formed, a single isolated colony was collected and inoculated in selective media. To ensure that all experiments were performed on bacteria coming from the same colony selected following streaking, fresh inocula for each bacterial strain were generated from the previous inocula. The bacterial concentration (CFU/mL) of each inoculum was determined in the mid-log growth phase by densitometry and CFU counting. The consistency between different experiments was by preparing fresh inocula before each experiment.

It should be noted that, while mainly known as an anaerobiotic bacteria, *C. acnes* is an aerotolerant anaerobe because it possesses enzymatic systems able to detoxify oxygen, allowing it to be sustained on the surface of the skin. As such, *C. acnes* can grow under conditions compatible with those experienced in skin.

### Preparation of scFOS

Solution composed of scFOS was prepared accordingly to Rossi et al*.*, 2005^16^. Briefly, depending on the experiment, scFOS were weighted and dissolved at a concentration of 20% (w/v) either in TSB or minimal medium [0.9% NaCl in sterile water + 0.003% of tryptic phosphate broth (TPB)1]. After dissolution, media sterilization was performed by autoclave.

### Bacteriostatic and bactericidal activity of scFOS

The ability of scFOS to inhibit bacterial strains growth (i.e. bacteriostatic activity) and/or to exert bactericidal activity was evaluated by measuring the Minimum Inhibitory Concentration (MIC) and Minimum Bactericidal Concentration (MBC). MIC was defined as the lowest concentration of an agent preventing bacterial growth, while MBC was lowest concentration of an antibacterial agent required to kill them. 10 × 10^6^ CFU/mL of each strain were exposed to increasing scFOS concentrations [from 0 to 15% (w/v)] under aerobic conditions, experienced by skin bacteria in vivo. The impact of scFOS concentrations on bacterial growth was evaluated at 0, 8 and 24 h and determined by colony counting. Once corrected for the dilution factor, bacterial growth was calculated as fold-change compared to the control (t = 0 h). Values higher than 1 indicated bacterial growth, equal to 1 a bacteriostatic activity (no bacterial growth), less than 1 a bactericidal activity.

### Impact of scFOS on bacterial growth kinetics

The ability of tested strains to metabolize scFOS to sustain their growth was explored. Following centrifugation and washing to eliminate the growth medium, 10 × 10^6^ CFU/mL were exposed to scFOS increasing concentrations (from 0 to 15%) in minimal medium at 37 °C for 24 h, under stirring and aerobic conditions. After 8 h and 24 h exposure, aliquots were collected and plated onto selective agar plates. Colonies were then counted and corrected for the appropriate dilution factor. Bacterial growth was expressed as fold-change compared to the bacterial load of the initial inoculum (t = 0 h).

### Competition between bacteria strains for scFOS

Bacterial ability to compete for scFOS as an energy source was evaluated by comparing *S. epidermidis vs C. acnes* and *S. epidermidis vs S. aureus*, at increasing scFOS concentrations (from 0 to 5%). After residual growth medium elimination, 10 × 10^6^ CFU/mL were exposed to scFOS increasing concentrations in minimal medium at 37 °C for 48 h, under agitation and aerobic conditions. At 0 h (initial bacterial load), 4, 8, 24 and 48 h, aliquots were sampled and seeded onto selective agar plates. For *S. epidermidis vs S. aureus* competition, seeding was performed on mannitol salt agar red phenol (MSARP), a differential medium able to discriminate between mannitol-fermenting (*S. aureus*) and non-mannitol fermenting (*S. epidermidis*) strains.

For *S. epidermidis *vs *C. acnes* competition, a differential colony counting strategy was applied. Collected aliquots were seeded on both MSARP and blood agar plates, and *C. acnes* calculates as the difference between colonies grown on blood and MSARP agar.

Once corrected for the appropriate dilution factor, bacterial growth was expressed as fold-change. The growth ratio was expressed as the ratio between the fold-change of *S. epidermidis* vs the fold-change of *C. acnes* and between the fold-change of *S. epidermidis* vs the fold-change of *S. aureus*.

### Bacterial strains competition for scFOS in a human reconstructed skin in vitro model (RHE)

The impact of scFOS on bacterial growth was also evaluated on a reconstructed human epidermis (RHE) in vitro model (Epiderm™, MatTek), characterized by a stratified epithelium and endowed with morpho-functional feature similar to the human epidermis. As such, this model can be effectively applied in bacterial strains adhesion, colonization and competition experiments. Briefly, the same number of bacteria (10 × 10^6^ CFU) for each strain was added to RHE surface. For competition experiments, *S. epidermidis* vs* C. acnes* and *S. epidermidis* vs* S. aureus* comparisons were evaluated. After adhesion for 3 h at 37 °C in a controlled-atmosphere incubator (5% CO_2_ and 85% relative humidity), RHE were thoroughly rinsed with Phosphate-Buffered Saline (PBS) to remove non-adhered bacteria. Adhered bacteria were then left to colonize the RHE for 18 h at 37 °C, and colonization was calculated as follows: Colonization (fold-change) = t_x_ (CFU/mL)/t0 (CFU/mL).

where, t_x_ was the number of CFU/mL following the colonization process and t0 was bacterial load (CFU/ml).

Bacteria-colonized RHE were exposed either to minimal medium only (negative control) and increasing scFOS concentrations. At 0, 8 and 24 h, scFOS-treated and scFOS-non treated RHE were rinsed with Hank’s Balanced Salt Solution (HBSS) and adhered bacteria detached with a modified scrub-wash approach. The obtained bacteria suspensions were serially diluted and plated according to previously mentioned specifications. Bacterial growth was expressed as fold-change compared to the initial bacterial load. The growth ratio was expressed as the ratio between the fold-change of *S. epidermidis vs* the fold-change of *C. acnes* and between the fold-change of *S. epidermidis vs* the fold-change of *S. aureus*.

### Statistical analysis

All statistical analyses were performed with OriginLab software. To determine if statistically significant differences between treatments were present, a t-test analysis was performed. All data were presented as mean ± standard deviation (SD) of three independent experiments. The differences between groups were considered significant at P < 0.05.

## Results

### Impact of scFOS on bacterial growth and survival

Before investigating the potential effect of scFOS as a prebiotic, its impact on *S. epidermidis*, *C. acnes* and *S. aureus* growth and survival was evaluated. No bacteriostatic or bactericidal effect of scFOS was observed on *S. epidermidis*, *C. acnes* and *S. aureus*, since a significant growth was observed at all tested concentrations compared to initial bacterial load (data not shown).

### Prebiotic activity of scFOS

The bacterial growth in minimal medium without scFOS was significantly limited compared to selective media (Table 1). The bacterial number of each bacterial strains at the different considered time-points is reported in Supplementary Table S1.

**Table 1:** Bacterial strains growth comparison between selective and minimal medium in absence of scFOS.

Bacterial strains	Time (h)	Selective medium	Minimal medium
*S. epidermidis*	0	1.0 ± 0.1	1.0 ± 0.1
	8	26.7 ± 0.5	7.1 ± 0.1
	24	23.7 ± 6.3	12.1 ± 5.3
*C. acnes*	0	1.0 ± 0.1	1.0 ± 0.2
	8	11.7 ± 1.0	0.3 ± 0.1
	24	0.3 ± 0.5	0.7 ± 0.0
*S. aureus*	0	1.0 ± 0.2	1.0 ± 0.2
	8	262.9 ± 11.5	9.4 ± 1.8
	24	141.5 ± 30.1	4.3 ± 1.3

Spread plate colony count, expressed as fold-change compared to the bacterial load of the initial inoculum, following exposure of *S. epidermidis, C. acnes* or *S. aureus* to selective and minimal medium for 8 and 24 h, in absence of scFOS. Results are expressed as mean ± SD.

**Figure 1:**
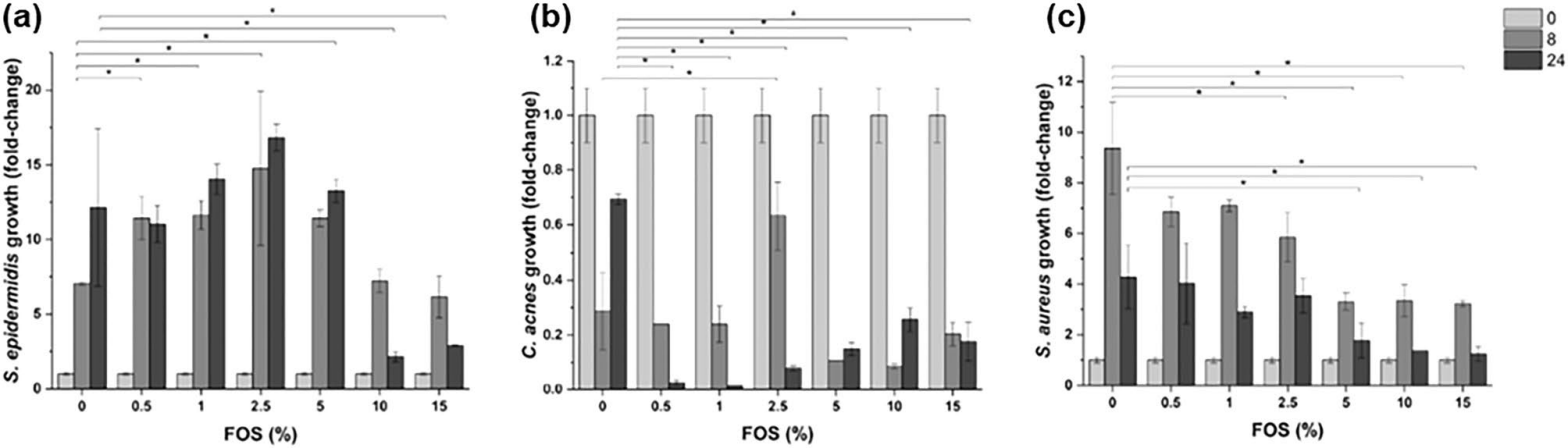
(**a**) *S. epidermidis*, (**b**) *C. acnes* and (**c**) *S. aureus* growth following exposure to different concentrations of scFOS at 0 (light grey column), 8 (grey column) and 24 h (dark grey column) in minimal medium in aerobic conditions. The results are expressed as fold-change compared to the initially inoculated quantity of each bacterium. To highlight the potential ability of the selected bacterial strains to sustain their growth by metabolizing scFOS, we exposed *S. epidermidis*, *C. acnes* and *S. aureus* at increasing scFOS concentration in minimal medium. *P < 0.05: significantly different from the value in absence of scFOS (0%) at the same time-point.

While *S. epidermidis* was able to exploit scFOS as an energy source, *S. aureus* and *C. acnes* were not able to metabolize scFOS (Fig. 1).

*S. epidermidis* significantly grew in the presence of 0.5 to 5% scFOS. At higher scFOS concentrations (10 and 15%), *S. epidermidis* growth was decreased. The presence of scFOS negatively impacted on *S. aureus* growth, since its presence decreased number of bacterial cells at 8 h and 24 h. Conversely from *S. aureus* population that was still able to grow in minimal conditions, *C. acnes* population growth was completely halted.

### Competition of bacterial strains for scFOS

**Figure 2:**
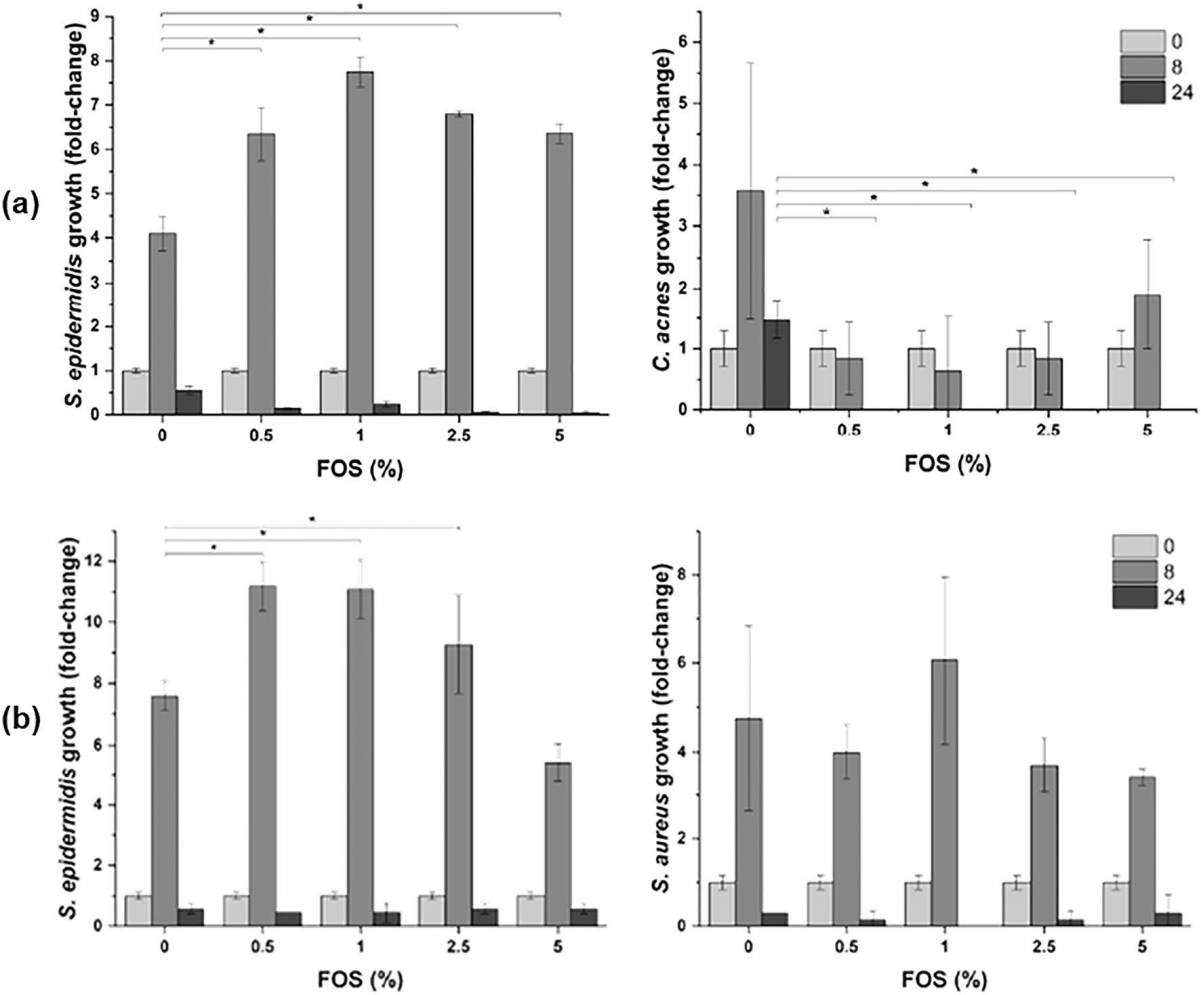
(**a**) *S. epidermidis* and *C. acnes* growth during competition and, (**b**) *S. epidermidis* and *S. aureus* growth during competition, in minimal medium following exposure to different concentrations of scFOS at 0 (light grey column), 8 (grey column) and 24 h (dark grey column) in aerobic conditions. The results are expressed as fold-change compared to the initially inoculated quantity of each bacterium. The effect of scFOS on the competition between *S. epidermidis* and *C. acnes* and *S. epidermidis* and *S. aureus* was investigated by inoculating the same amount of the different bacterial strains in minimal medium, in presence of increasing concentration of scFOS (from 0 to 5%). *P < 0.05: significantly different from the value in absence of scFOS (0%) at the same time-point.

Competition for scFOS as energy source was evaluated by comparing *S. epidermidis* and *C. acnes* and *S. epidermidis* and *S. aureus*. *S. epidermidis* and *C. acnes* competition for scFOS confirmed the previously observed results, with the increased *S. epidermidis* growth at all tested scFOS concentrations and the inability of *C. acnes* to grow (Fig. 2a). The *S. epidermidis*/*C. acnes* growth ratio reached a maximum at 1% scFOS after 8 h incubation, while CFU of both strains decreased after 24 h probably due to depletion of nutrients. The spread plate colony counts for the competition between *S. epidermidis* and *C. acnes* are reported in Supplementary Table S2.

*S. epidermidis* and *S. aureus* competition showed an increase in *S. epidermidis* growth in the range between 0.5 to 2.5% scFOS and an scFOS-independent growth of *S. aureus* (Fig. 2b).

The *S. epidermidis* and *S. aureus* growth ratio confirmed the ability of *S. epidermidis* to grow by using scFOS at the expense to the detriment of the growth of *S. aureus*, with the highest stimulation obtained for the smallest scFOS concentration (0.5%) (Table 2). The spread plate colony counts for the competition between *S. epidermidis* and *S. aureus* are reported in Supplementary Table S3.

**Table 2:** Competition of bacterial strains for scFOS.

scFOS (%)	*S. epidermidis*/*C. acnes* growth ratio		*S. epidermidis*/*S. aureus* growth ratio	
	8 h	24 h	8 h	24 h
0	1.1	–	1.6	2.0
0.5	7.8	–	2.8	5.0
1	12.8	–	1.8	–
2.5	8.5	–	2.5	6.0
5	3.4	–	1.6	2.0

Spread plate colony count of *S. epidermidis* and *C. acnes* competition, as well as *S. epidermidis* and *S. aureus* competition for scFOS at different concentrations in minimal medium, expressed as fold-change compared to the initial bacterial load. Results are expressed as mean ± SD. – value non calculated due to one 0 value.

### Competition of bacterial strains for scFOS on human reconstructed in vitro skin model

The impact of scFOS on the growth of the three strains was also evaluated in reconstructed human epithelium (RHE). Firstly, the colonization of bacterial strains was evaluated as a result of their adhesion and colonization. *S. epidermidis* presented the highest colonization potential (1721.7 ± 160.1 fold-change), while *S. aureus* and *C. acnes* had colonization potentials of 33.4 ± 6.6 fold-change and 2.2 ± 0.1 fold-change, respectively. The growth of bacterial strains on in vitro skin model RHE was evaluated exposing RHE to 0.5% and 1% scFOS up to 24 h. No significant increase in *S. epidermidis* population was observed after 8 h, while a significant increase was highlighted following 0.5% scFOS for 24 h (Table 3). *C. acnes* showed the highest growth among tested bacteria, and *S. aureus* population increased significantly over time at both tested scFOS concentrations (Table 3). The spread plate colony counts for the evaluation of selected bacterial strains growth on RHE in presence of increasing concentration of scFOS are reported in Supplementary Table S4.

**Table 3:** Bacterial strains growth on reconstructed human epithelium (RHE).

scFOS (%)	*S. epidermidis*			*C. acnes*			*S. aureus*		
	0 h	8 h	24 h	0 h	8 h	24 h	0 h	8 h	24 h
0	1.0	0.6	0.7	1.0	1.5	51.4	1.0	2.5	1.6
0.5	1.0	0.6	1.6	1.0	8.4	67.5	1.0	7.9	2.4
1	1.0	0.5	1.0	1.0	0.5	18.3	1.0	12.2	18.1

Spread plate colony count of *S. epidermidis*, *C. acnes* and *S. aureus* following RHE colonization and exposure to different concentrations of scFOS for 0, 8 and 24 h, expressed as fold-change compared to the initial bacterial load.

The *S. epidermidis* and *C. acnes* competition for scFOS showed a positive effect on *S. epidermidis* after 8 h exposure to 0.5% prebiotic, which was no longer visible after 24 h (Table 4). At 1% scFOS, the ratio was reversed with *C. acnes* that grew faster than *S. epidermidis* (ratio < 1) both after 8 and 24 h exposure. However, between 8 and 24 h exposure to 1% scFOS, *C. acnes* population decreased while *S. epidermidis* population increased, highlighted by a *S. epidermidis*/*C. acnes* ratio that tended to increase, from 0.3 to 0.9. The spread plate colony counts for the competition between *S. epidermidis* and *C. acnes* for scFOS at the human reconstructed epidermis level are reported in Supplementary Table S5.

**Table 4:** Competition of bacterial strains for scFOS on human reconstructed epithelium (RHE).

scFOS (%)	*S. epidermidis*/*C. acnes* growth ratio		*S. epidermidis*/*S. aureus* growth ratio	
	8 h	24 h	8 h	24 h
0	3.8	0.6	0.2	–
0.5	2.7	0.4	0.3	–
1	0.3	0.9	0.2	–

Spread plate colony count of *S. epidermidis* and *C. acnes* competition for scFOS as well as *S. epidermidis* and *S. aureus* competition for scFOS at different concentrations on RHE, expressed as fold-change compared to the bacterial load at t = 0. Results are expressed as mean ± SD of three independent experiments. – value non calculated due to one 0 value.

Regarding *S. epidermidis* vs *S. aureus* competition, results indicated that *S. aureus* outgrew *S. epidermidis* at 8 h, with no impact of scFOS exposure (Table 4). The spread plate colony counts for the competition between *S. epidermidis* and *S. aureus* for scFOS at the human reconstructed epidermis level are reported in Supplementary Table S6.

## Discussion

The skin microbiota represents a largely unexplored but rapidly emerging field in the personal care industry, as demonstrated by the increasing number of formulations incorporating ingredients which might impact the skin microbiota balance. Among them, prebiotics can promote the growth of microorganisms essential for the well-being of the skin, simultaneously preventing that of harmful microorganisms.

Therefore, the current research evaluated the effects of scFOS containing skin formulation on the skin microbiota composition through in vitro model.

For the hazard identification for EU CLP Regulation (1272/2008), the irritation potential of scFOS has been previously characterised. The skin irritation and corrosion potential were assessed using a RHE model. The conclusion was that scFOS did not require to be classified as skin irritant or even skin corrosive. In addition, a local lymph node assay was performed with the substance, and it was confirmed that there was no skin sensitization potential. This was completed with an isolated chicken eye test which concluded that scFOS did not require classification for eye irritation or serious eye damage. Altogether, these information provided reassuring data information on the ability for scFOS to be used safely as a cosmetic ingredient, before going further with our study targeting the skin microbiota composition.

Three bacterial strains, *S. epidermidis*, *C. acnes* and *S. aureus*, were selected as representative of the human skin microbiota. While *S. epidermidis* is identified as a normal member of skin microbiota, both *C. acnes* and *S. aureus* are opportunistic pathogens that can be involved in the onset and development of skin pathologies. In particular, *C. acnes* is considered to be one of the causes of acne vulgaris, while *S. aureus* can be responsible for complications and inflammation during psoriasis and skin wounds^17^. In the light of obtained results, scFOS was not endowed with bacteriostatic or bactericidal activity on the three tested bacterial strains, since a significant growth was observed at all tested concentrations compared to initial bacterial load.

Once determined that scFOS did not inhibit the growth of tested bacterial strains (i.e. bacteriostatic activity) or kill them (i.e. bactericidal activity), the role of scFOS as energy source for *S. epidermidis*, *C. acnes* and *S. aureus* was evaluated. To ensure that bacterial growth was directly dependent on scFOS metabolization, the experiments were performed in minimal medium, able to sustain bacterial survival with limiting effects on their growth. Results indicated that scFOS acted as a prebiotic for *S. epidermidis* from 0.5 to 5%, promoting and sustaining its growth up to 24 h. On the contrary, no scFOS prebiotic effect was observed for *C. acnes* and *S. aureus*, demonstrating that scFOS selectively enhanced the growth of *S. epidermidis*, considered as a beneficial strain for skin microbiota homeostasis.

A growing body of evidence clearly demonstrated that skin microbiota is an extremely complex community. The delicate equilibrium underlying the homeostasis of this community is dependent on the intricate network of interactions between microorganisms. As such, slight variations in these interactions may lead to skin microbiota dysbiosis, possibly leading to skin diseases^8^. For this reason, it was also fundamental to evaluate the competition between bacteria strains for scFOS use as an energy source. Since *C. acnes* and *S. aureus* are considered as potentially pathogenic bacteria, conversely to *S. epidermidis*, the competition for scFOS between *S. epidermidis vs C. acnes* and *S. epidermidis vs S. aureus* was investigated. Results demonstrated that scFOS selectively enhanced *S. epidermidis* growth at the expenses of both *C. acnes* and *S. aureus*, confirming the prebiotic activity of scFOS on skin microbiota.

Previous studies highlighted some competition between our studied strains. *S. epidermidis* has been shown to antagonize *C. acnes*, highlighting its therapeutic potential against acne^18^. On the other hand, clinical isolates of *S. epidermidis* inhibited *S. aureus* biofilm formation^19^. Indeed, *S. epidermidis* produced several molecules, as phenol-soluble modulins (PSMs) with antimicrobial functions, able to interfere with the growth of pathogens, such as *S. aureus*^3,20^. In this way, *S. epidermidis* may help prime the immune system and enhance innate immunity^5^. Thereby, by selectively favouring beneficial bacteria growth over potentially pathogenic bacteria, the use of scFOS in cosmetic formulations may be instrumental in recovering skin homeostasis with potential benefits on skin health.

Therefore, the impact of scFOS on these different strains using a reconstructed human epithelium (RHE) was assessed. *S. epidermidis* showed the highest colonization potential, resulting from the combination of its adhesive and colonization abilities, with *C. acnes* being the least inclined to colonize the skin. As single strain on RHE, *S. epidermidis* only growed when exposed to scFOS for 24 h. On the contrary, *S. aureus* population growed significantly over time following exposure to both scFOS concentrations, similarly for *C. acnes* but only at the dose of 0.5% scFOS. The high growth of *C. acnes* on RHE can be explained by the availability of fatty acids on RHE that could have propelled the growth of *C. acnes* which is mainly a fatty acid-metabolizing bacteria^5^. Competition experiments showed that *S. epidermidis* growth was higher than *C. acnes* up to 8 h. The ratio was reversed at longer exposure time (24 h). Noteworthy, a decreasing trend for *C. acnes* growth was observed, while *S. epidermidis* population tended to increase with 1% scFOS suggesting a long-term effect of scFOS on *S. epidermidis* growth.

Altogether, the results demonstrated that scFOS could represent an interesting strategy to rebalance the composition of the skin microbiota by inhibiting the growth of potentially pathogenic bacteria and, at the same time, preserving the growth of beneficial bacteria. There was a lack of literature on the effects of prebiotics on skin microbiota and health. One study showed that konjac glucomannan hydrolysates (GMH) can inhibit the growth of undesirable bacteria such as *C. acnes *in vitro^7^. Plant extracts like Ginseng or Black currant could also inhibit the inflammation‐causing bacterium *C. acnes* without affecting beneficial species like coagulase‐negative *Staphylococci*^21^. In addition, 5% of gluco-oligosaccharides can be used to control the growth of *S. aureus *in vitro^22^, similarly to GOS exposure in vivo for 6 weeks^23^.

Our study is the first to indicate that scFOS, a well-recognized prebiotic ingredient for gut microbiota, can also effectively modulate the composition of the skin microbiota by inhibiting the growth of potentially pathogenic bacteria and, at the same time, promoting the growth of beneficial bacteria. Therefore, scFOS is an efficient ingredient to preserve or restore the natural individual balance of the skin microbiota and it can be used easily in skincare applications. Its effects on the structure and functionality of the skin are still unknown and should be investigated in further studies, in particular through clinical studies.

## Supplementary Information

Supplementary Tables.

## Data Availability

The datasets generated during the current study are not publicly available due to Patent Application but are available from the corresponding author on reasonable request.
