# Supplementary material for: Effects of short chain fructo-oligosaccharides on selected skin bacteria
Source: Sci Rep. 2022 Jun 11;12:9702. doi: 10.1038/s41598-022-13093-5 (PMC9188601; doi:10.1038/s41598-022-13093-5)
Supplement: Supplementary file 1 — Supplementary Tables. [file 41598_2022_13093_MOESM1_ESM.pdf]

Effects of short chain fructo-oligosaccharides on selected skin bacteria

Cindy Le Bourgot[1*], Claire Meunier[1], Elisa Gaio[2], Vincent Murat[1], Marta Micheletto[2], Erik Tedesco[2], Federico Benetti[2]

[1] Tereos, R&D department, Rue de Senlis, 77290 Moussy-Le-Vieux, France ;

[2] ECSIN-ECAMRICERT SRL Laboratory, Corso Stati Uniti, 4 – 35127 Padova, Italy.

[*] Corresponding author: cindy.lebourgot@tereos.com; Tel.: +33 698 385 852

SUPPLEMENTARY TABLES

Supplementary Table S1. Bacterial strains growth comparison between selective and minimal medium in absence of scFOS. Spread plate colony count, expressed as CFU/mL, following exposure of *S. epidermidis, C. acnes* or *S. aureus* to selective and minimal medium for 8 and 24h, in absence of scFOS.

Bacterial Strains	Time (h)	Selective Medium	Minimal Medium
S. epidermidis	0	7.8×10^{6}	1.3×10^{6}
	8	2.1×10^{8}	8.9×10^{6}
	24	1.8×10^{8}	1.5×10^{7}
C. acnes	0	2.9×10^{8}	3.0×10^{7}
	8	3.4×10^{9}	0.9×10^{7}
	24	1.0×10^{8}	2.1×10^{7}
S. aureus	0	1.2×10^{7}	0.1×10^{7}
	8	3.1×10^{9}	1.2×10^{7}
	24	1.7×10^{9}	0.5×10^{7}

Supplementary Table S2. Competition of *S. epidermidis* and *C. acnes* for scFOS. Spread plate colony count, expressed as CFU/mL, following exposure of *S. epidermidis* and *S. aureus* to increasing concentration of scFOS (0 to 5 %) in minimal medium and aerobic conditions.

	S. epidermidis (CFU/mL)		*C. acnes* (CFU/mL)	
scFOS (%)	8h	24h	8h	24h
0	4.6×10^{7}	6.2×10^{6}	1.7×10^{6}	–
0.5	7.1×10^{7}	1.6×10^{6}	4.0×10^{5}	–
1	8.7×10^{7}	2.7×10^{6}	3.0×10^{5}	–
2.5	7.6×10^{7}	6.0×10^{5}	4.0×10^{5}	–
5	7.1×10^{7}	5.0×10^{5}	9.0×10^{5}	–

Supplementary Table S3. Competition of *S. epidermidis* and *S. aureus* for scFOS. Spread plate colony count, expressed as CFU/mL, following exposure of *S. epidermidis* and *S. aureus* to increasing concentration of scFOS (0 to 5 %) in minimal medium and aerobic conditions.

	***S. epidermidis* (CFU/mL)**		***S. aureus* (CFU/mL)**	
**scFOS (%)**	**8h**	**24h**	**8h**	**24h**
**0**	6.7×10^{6}	5.0×10^{5}	3.2×10^{6}	2.0×10^{5}
**0.5**	9.9×10^{6}	4.0×10^{5}	2.7×10^{6}	1.0×10^{5}
**1**	9.8×10^{6}	4.0×10^{5}	4.1×10^{6}	–
**2.5**	8.2×10^{6}	5.0×10^{5}	2.5×10^{6}	1.0×10^{5}
**5**	4.8×10^{6}	5.0×10^{5}	2.3×10^{6}	2.0×10^{5}

Supplementary Table S4. Bacterial strains growth on reconstructed epidermis in presence of scFOS. Spread plate colony count, expressed as CFU/mL, following exposure of *S. epidermidis, C. acnes* or *S. aureus* increasing scFOS concentrations in minimal medium for 0, 8 and 24h.

	***S. epidermidis* (CFU/mL)**			***C. acnes* (CFU/mL)**			***S. aureus* (CFU/mL)**		
**scFOS (%)**	**0h**	**8h**	**24h**	**0h**	**8h**	**24h**	**0h**	**8h**	**24h**
**0**	1.6×10^{8}	9.8×10^{7}	1.2×10^{8}	3.5×10^{5}	5.1×10^{5}	1.8×10^{7}	4.9×10^{6}	1.2×10^{7}	7.9×10^{6}
**0.5**	3.9×10^{8}	2.5×10^{8}	6.3×10^{8}	3.3×10^{5}	2.7×10^{6}	2.2×10^{7}	3.1×10^{6}	2.4×10^{7}	7.3×10^{6}
**1**	1.8×10^{8}	1.0×10^{8}	1.9×10^{8}	1.3×10^{6}	6.8×10^{5}	2.4×10^{7}	2.4×10^{6}	2.9×10^{7}	4.2×10^{7}

Supplementary Table S5. Competition of *S. epidermidis* and *C. acnes* for scFOS on human reconstructed epidermis. Spread plate colony count, expressed as CFU/mL, following exposure of *S. epidermidis* and *C. acnes* to increasing concentration of scFOS (0 to 1 %) in minimal medium, aerobic conditions and on human reconstructed epidermis.

	***C. acnes* (CFU/mL)**	***S. epidermidis* (CFU/mL)**	***C. acnes* (CFU/mL)**	***S. epidermidis* (CFU/mL)**	***C. acnes* (CFU/mL)**	***S. epidermidis* (CFU/mL)**
**scFOS (%)**	**0h**	**0h**	**8h**	**8h**	**24h**	**24h**
**0**	2.2×10^{5}	1.5×10^{8}	5.0×10^{4}	1.3×10^{8}	5.0×10^{5}	2.0×10^{8}
**0.5**			5.0×10^{4}	9.3×10^{7}	1.0×10^{6}	2.8×10^{8}
**1**			5.0×10^{4}	1.1×10^{8}	5.0×10^{5}	3.0×10^{8}

Supplementary Table S6. Competition of *S. epidermidis* and *S. aureus* for scFOS on human reconstructed epidermis. Spread plate colony count, expressed as CFU/mL, following exposure of *S. epidermidis* and *S. aureus* to increasing concentration of scFOS (0 to 1 %) in minimal medium, aerobic conditions and on human reconstructed epidermis.

	***S. aureus* (CFU/mL)**	***S. epidermidis* (CFU/mL)**	***S. aureus* (CFU/mL)**	***S. epidermidis* (CFU/mL)**	***S. aureus* (CFU/mL)**	***S. epidermidis* (CFU/mL)**
**scFOS (%)**	**0h**	**0h**	**8h**	**8h**	**24h**	**24h**
**0**	8.3×10^4	2.0×10^7	6.0×10^5	2.9×10^7	–	–
**0.5**			1.5×10^5	1.1×10^7	–	–
**1**			1.6×10^6	6.4×10^7	–	–
